# Synthesis of Capped A^II^B^VI^ Nanoparticles for Fluorescent Biomarkers

**DOI:** 10.1186/s11671-016-1300-5

**Published:** 2016-02-11

**Authors:** Galyna Rudko, Volodymyr Fediv, Igor Davydenko, Evgen Gule, Olena Olar, Andrii Kovalchuk

**Affiliations:** V. Lashkaryov Institute of Semiconductor Physics of National Academy of Sciences of Ukraine, 45, Pr. Nauky, Kiev, 03028 Ukraine; Department of Biophysics and Medical Informatics, Bukovinian State Medical University, 42 Kobylyanska st., 58000 Chernivtsi, Ukraine

**Keywords:** CdS, Nanoparticles, Biomarker, Fluorescence, Mercaptoethanol

## Abstract

The conditions for growing CdS nanoparticles suitable for the visualization of biological tissues were theoretically studied and experimentally checked. The optimal ranges for pH values and precursors’ concentrations were determined. The applicability of the mercaptoethanol-capped nanoparticles for in vitro luminescence visualization of several cellular forms in histological specimens of human placenta has been proven.

## Background

In the past decades, considerable progress has been achieved in designing novel tools for various fields of biomedical diagnostics by the combination of colloidal nanoparticles (NPs) with biomolecules or cells (for the review, see, e.g., [1–4]). In the field of bio-imaging, NPs demonstrate several attractive advantages as compared to traditional organic dyes such as broad absorption, tunable emission spectra, better photostability, and brighter contrast. Therefore, the traditional biomedical diagnostics based on the dyeing of histological specimens with various organic dyes are now shifting to NP-based methods in order to achieve improved stability of the optical image intensity.

In spite of numerous works done on the NP growth, there are still a lot of challenges to be overcome in this field. As a rule, the synthesis-related papers provide the information on the quantitative characteristics of reactants and other growth conditions while the substantiation of the choice of these values is, as a rule, omitted. Therefore, the continuous research work is in progress in order to give a clear-cut understanding of the optimized conditions for the synthesis of NPs.

Another important aspect of the recent research in the nano-imaging field is the search for advanced functionalization of NPs for bio-application by applying various surface coverings that play a dual role of stabilizing colloid and preventing possible damaging influence of inorganic NPs on the biological samples.

Thus, the aims of the present paper were to analyze in detail the conditions in the growth solution at variable precursors’ concentrations and pH values and to find out the optimal ranges of these parameters for successful NP synthesis, to apply the estimated parameters to the fabrication of NPs, to cover the surface of CdS with thiol-containing groups, and to check the applicability of the synthesized NPs to biomedical studies.

## Methods

### Procedure of Nanoparticle Synthesis

The multi-step procedure of colloidal synthesis was used. The first step was the preparation of the starting growth medium — the water solution of capping molecules that served for restricting NP sizes, preventing NP coagulation, and passivating the surface. As the synthesis targeted the production of NPs for biological applications, we had a concern in avoiding the known toxicity of cadmium ions which tend to bind to the thiol groups of some molecules in cells and cause significant damage [5]. In the present study, we used the bifunctional molecules 2-mercaptoethanol [HS-(CH_2_)_n_-OH] which contain thiol (–SH) groups. The binding of 2-mercaptoethanol occurs through this sulfur group at the molecule terminus, which adheres to Cd ions in NPs. Thus, this prevents the binding of Cd ions to the species in the surrounding biological tissue. The hydroxyl end of 2-mercaptoethanol provides water solubility. Moreover, because of –OH groups, these molecules may be reactive to biomolecules due to the intermolecular hydrogen bonding.

The second synthesis step was the addition of CdCl_2_ precursor to the solution of 2-mercaptoethanol. During all following steps, the precursors of NPs (Na_2_S and CdCl_2_) were added to the solution alternatively until the concentrations met the demands deduced in the next section. All synthesis steps were done at ambient conditions.

The synthesis parameters, namely the precursors’ concentrations and pH values, were adjusted in accordance with the results of our analysis of the chemical reaction probabilities in multi-component solution (see the next section). We conducted several growth procedures using several starting solutions with different pH that were chosen within the optimal parameter range (pH = 4–7). The experimental results show that only the colloidal solutions obtained at the pH of the starting solution in the range 4–5.5 are stable.

### Characterization Techniques

The light-emitting properties of thus synthesized NPs were studied at the excitation with the 375-nm beam of the diode laser NS-375L-5RLO using the MDR-23 monochromator with FEU-100 photomultiplier for registration.

The images of the fixed biological tissues were obtained using the fluorescent microscope LUMAM-P-8 and digital camera Olympus C740UZ.

## Results and Discussion

### Analysis of Synthesis Conditions

To achieve successful NP fabrication, one has to adjust the composition of a multi-component solution in such a way that the reaction of CdS growth would dominate while formation of undesirable compounds would be much less probable. In what follows, we theoretically analyze the influence of pH and precursors’ concentrations on the content of various ion species in the growth solution, which are the prerequisite of NP formation, and determine the ranges of these parameters where NP formation is favored.

Both cadmium and sulfur, which are the “building bricks” for NPs, can occur in the growth solution in various ionic and molecular forms. The availability or deficiency of necessary species strongly influences the reaction. The content of ionic and molecular species can be estimated using the balance equations.

The balance equation for cadmium can be written as:

1 $${C}_0=\left[{\mathrm{Cd}}^{2+}\right]+\mathrm{C}\mathrm{d}{\left(\mathrm{O}\mathrm{H}\right)}^{+}+\mathrm{C}\mathrm{d}{\left(\mathrm{O}\mathrm{H}\right)}_2^0+\mathrm{C}\mathrm{d}{\left(\mathrm{O}\mathrm{H}\right)}_3^{-},$$

Here, *C*_0_ is the total concentration of Cd-related species in the solution, and [Cd^2+^], [Cd(OH)^+^], [Cd(OH)^0^_2_], and [Cd(OH)^−^_3_] are the concentrations of Cd-related species.

Note that, for simplicity, only the hydroxo complexes of $\mathrm{C}\mathrm{d}{\left(\mathrm{O}\mathrm{H}\right)}_n^{2-n}$ type were taken into account in (1).

Using the hydrolysis constant for the ions Cd^2+^ [6], the following equation for the mole fraction of the product of CdCl_2_ salt hydrolysis was obtained:

2 $${\alpha}_1=\frac{\left[{\mathrm{C}\mathrm{d}}^{2+}\right]}{{\mathrm{C}}_{{\mathrm{C}\mathrm{d}\mathrm{Cl}}_2}}=\frac{1}{1+\frac{k_1}{\left[{\mathrm{H}}^{+}\right]}+\frac{k_1{k}_2}{{\left[{\mathrm{H}}^{+}\right]}^2}+\frac{k_1{k}_2{k}_3}{{\left[{\mathrm{H}}^{+}\right]}^3}}$$

3 $${\alpha}_2=\frac{k_1\cdot {\alpha}_1}{\left[{\mathrm{H}}^{+}\right]}$$

4 $${\alpha}_3=\frac{k_1\cdot {k}_2\cdot {\alpha}_1}{{\left[{\mathrm{H}}^{+}\right]}^2}$$

5 $${\alpha}_4=\frac{k_1\cdot {k}_2\cdot {k}_3\cdot {\alpha}_1}{{\left[{\mathrm{H}}^{+}\right]}^3}$$

where ${C}_{{\mathrm{CdCl}}_2}$ is the concentration of CdCl_2_ salt; *k*_1_, *k*_2_, and *k*_3_ are the hydrolysis constants; *α*_1_, *α*_2_, *α*_3_, and *α*_4_ are the mole fractions of the products of CdCl_2_ salt hydrolysis—Cd^2+^, Cd(OH)^+^, Cd(OH)^0^_2_, and Cd(OH)^−^_2_, respectively; and [H^+^] is the concentration of H^+^.

**Fig. 1:**
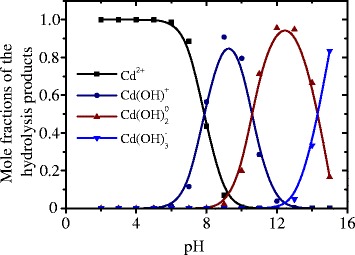
Dependences of the mole fractions of the products of CdCl_2_ salt hydrolysis—Cd^2+^, Cd(OH)^+^, Cd(OH)^0^_2_, and Cd(OH)^−^_2_—vs. pH of the solution

Based on these formulae, we calculated the dependences of the mole fractions of various hydrolysis products of CdCl_2_ salt vs. pH of the growth solution (Fig. 1). It is seen that at pH < 7.8 the mole fraction of Cd^2+^ ions is more than 50 % while at higher values of pH the hydroxo complexes are preferably formed, namely Cd(OH)^+^, Cd(OH)^0^_2_, and Cd(OH)^−^_2_ prevail in the ranges рН = 7.8–10.5, рН = 10.5–14.3, and рН > 14.3, respectively.

To analyze the content of various sulfur-related species vs. the pH, we used the balance equation for sulfur (the notations are of the same type as in (1)):

6 $${C}_0={\mathrm{H}}_2\mathrm{S}+{\mathrm{H}\mathrm{S}}^{-}+{\mathrm{S}}^{2-}$$

The mole fractions of Na_2_S hydrolysis products (S^2−^, HS^−^, and H_2_S) were calculated using the following formulae:

7 $$\alpha \left({\mathrm{S}}^{2-}\right)=\frac{k_1{k}_2}{k_1{k}_2+\left[{\mathrm{H}}^{+}\right]{k}_1+{\left[{\mathrm{H}}^{+}\right]}^2}$$

8 $$\alpha \left({\mathrm{H}\mathrm{S}}^{-}\right)=\frac{k_1\cdot \left[{\mathrm{H}}^{+}\right]}{k_1{k}_2+\left[{\mathrm{H}}^{+}\right]{k}_1+{\left[{\mathrm{H}}^{+}\right]}^2}$$

9 $$\alpha \left({\mathrm{H}}_2\mathrm{S}\right)=\frac{{\left[{\mathrm{H}}^{+}\right]}^2}{{\left[{\mathrm{H}}^{+}\right]}^2+{k}_1\left[{\mathrm{H}}^{+}\right]+{k}_1{k}_2}$$

The notations in (7)–(9) are of the same type as in (2)–(5).

**Fig. 2:**
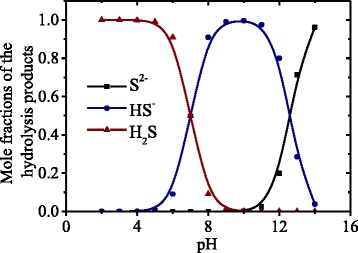
Dependences of the mole fractions of the products of Na_2_S salt hydrolysis (S^2−^, HS^−^, and H_2_S) vs. pH of the solution

Based on (7)–(9), the dependences of mole fractions of various sulfur-containing species vs. pH were calculated. They are shown in Fig. 2. It is seen that at the lowest pH values, the molecular species H_2_S dominate; at higher pH, the ions HS^−^ are also present in the solution; and at the highest pH values, the ions S^2−^ dominate.

We have also analyzed one more chemical reaction that can interfere with CdS NP formation. It is known that, besides the reaction of hydro-complex formation, the chloride complexes [CdCl_n_]^2 − n^ can also be formed [7]. The balance equation for Cd ions is:

10 $${C}_{{\mathrm{CdCl}}_2}={\left[\mathrm{C}\mathrm{d}\right]}^{2+}+\left[{\mathrm{CdCl}}^{+}\right]+\left[{\mathrm{CdCl}}_2^0\right]+\left[{\mathrm{CdCl}}_3^{-}\right]+\left[{\mathrm{CdCl}}_4^{2-}\right]$$

**Fig. 3:**
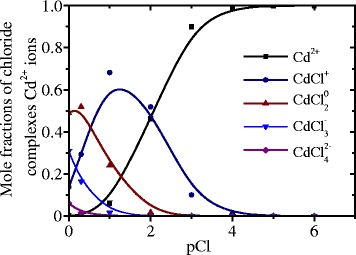
Dependences of the concentrations of chloride complexes of Cd vs. concentration of Cl^−^ ions

Using the constants of the cadmium chloride complex stability [8], we have calculated the mole fractions of complex ions vs. concentration of Cl^−^ ions (Fig. 3). Based on this analysis, we conclude that Cd^2+^ ions prevail in the solution until their concentration is less than 0.05 mol/dm^3^.

An important issue for optimization of the synthesis process is preventing exhaustion of cadmium content via precipitation of Cd-involving hydroxides. We have calculated the minimal pH values, at which Cd(ОН)_2_ precipitation starts, pH_min_. Based on the solubility products for Cd(OH)_2_ [9], we have obtained the dependence of pH_min_ on the concentration of the salt in the system CdCl_2_-Na_2_S-H_2_O:

11 $${\mathrm{pH}}_{\min }=7.17-\frac{1}{2} \lg \left[{\mathrm{Cd}}^{2+}\right]$$

**Fig. 4:**
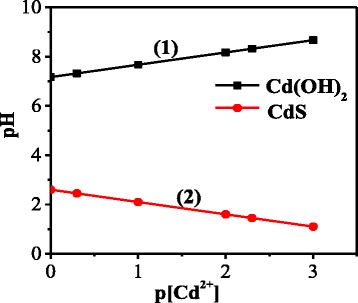
Dependencies of pH_min_ (*1*) and pH^S^_min_ (*2*) on the concentration of Cd^2+^ ions

The corresponding dependence is shown in Fig. 4.

Similar analysis was done to find out the minimal pH values that ensure formation of sulfides. Based on the solubility products of CdS [9], the dependence of pH^S^_min_ on the concentration of Cd^2+^ ions in the solution was derived:

12 $${\mathrm{pH}}_{\min}^S=2.6-\frac{1}{2}p\left[{\mathrm{Cd}}^{2+}\right]$$

The latter dependence is also shown in Fig. 4. The region between these two curves is the parameter range where only CdS is formed while Cd(ОН)_2_ is not.

Thus, to adjust the conditions for CdS NP synthesis, one has to achieve a compromise among all the deduced demands to the growth parameters; namely, based on the analysis of the hydrolysis of Cd-containing salt, the optimal values of the parameters for the successful NP growth are pH = 4–7, and Cd^2+^ ion concentration must be less than 0.05 mol/dm^3^. Based on the analysis of the hydrolysis of S-containing salt, we concluded that, within these ranges of pH and Cd salt concentration, the growth of NPs occurs via the reaction with HS^−^ ions. Note that the demands as to the concentration of sulfur-containing species are less strict because one can choose either sulfur-deficient growth or the growth with the excess of sulfur.

**Fig. 5:**
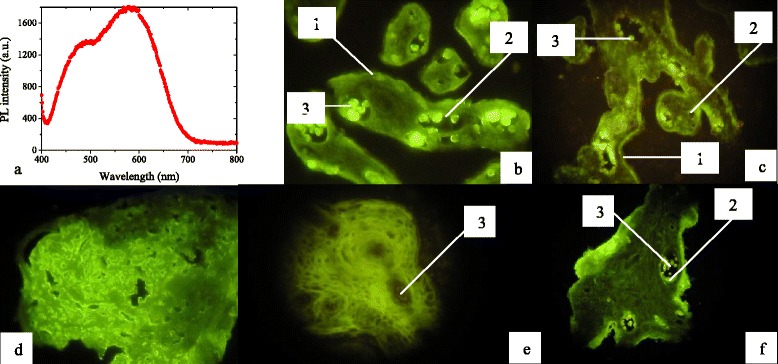
PL and microscopic images: typical photoluminescence spectrum of the colloidal solution of mercaptoethanol-coated CdS NPs (**a**) and microscopic images (**b**–**f**) of histological sections of placenta tissue (fixed and preserved) visualized using mercapto-coated NPs: **b** unaltered human placental terminal villi, **c** unaltered human intermediate villus with dichotomy to terminal villi, **d** intervillous fibrinoid, **e** human intermediate stem villus with artery and vena and rough collagen, and **f** human intermediate villus with damaged surface with fibrinoid. The identified forms are *1*—trophoblast, *2*—endotheliocyte, and *3*—erythrocytes

NPs that were grown by the route described above demonstrated bright emission under UV excitation. The typical spectrum of the colloidal solution of CdS NPs capped with mercaptoethanol is shown in Fig. 5a.

### Application of the Synthesized NPs for Fixed Tissue Visualization

We have tested the applicability of the synthesized colloidal solutions of CdS NPs for biomedical purposes, namely for the visualization of the fixed biological tissues. The specimens used were the histological sections of human placenta at the 32–40 weeks of pregnancy term. The thickness of microtome slices was 5 μm. The placenta was chosen as an experimental object because it contains certain structures of the chorial tree that can be easily identified.

The tissues were treated using the technique that is described in [10]. This fixation method introduces minimal changes into the structural and chemical properties of the tissues with a possibility of their prolonged conservation in paraffin blocks. De-embedding of histological sections was performed by means of a standard technique, using xylol, ethanol, and water.

For the dyeing of the histological sections with NPs, the sample was completely covered with a colloidal solution. The exposure time varied in the range 5–10 min. After the exposure, the sections were washed in the distilled water and then dried.

Figure 5b–f shows the fluorescence microscopic images of the human placenta histological sections. Some structures of the chorial tree can be distinguished and identified based on well-documented morphological features of the chorial villi and extravillous structures of the placenta with 40-week pregnancy term [11].

The most intense luminescence is observed in the maternal erythrocytes and fetal erythrocytes, as well as in the intervillous fibrinoids. Less intense, but still well defined, luminescence is seen in the syncytiotrophoblast and endotheliocytes of the blood vessels of the chorial villi which are preliminarily identified as stromal cells, taking into account the dimensions, forms, and the specific characteristics of the location (most reliably, with due regard for the term of pregnancy—fibroblasts).

## Conclusions

The theoretical analysis of the reactions in the growing solution for CdS NPs is carried out, and the optimal ranges of several parameters for successful NP synthesis were calculated. The experimental application of these conditions demonstrated that the theoretically estimated pH parameter range should be reduced in view of instability of colloids obtained at high pH values.

Thus, the optimal values of the parameters are pH = 4–5.5, and the concentration of Cd salt is <0.05 mol/dm^3^. The concentration of sulfur salt can be varied depending on the choice of sulfur-deficient or sulfur-excess growth.

The NPs grown in accordance with the above conditions were applied for visualization of fixed and preserved histological sections of human placenta. Various cellular forms, such as maternal erythrocytes and fetal erythrocytes, syncytiotrophoblasts, and endotheliocytes, were identified.
